# Retraction: Case Report: Coronavirus disease 2019 (COVID-19) modified RNA vaccination-induced adult-onset still's disease with fulminant myocarditis as initial presentation

**DOI:** 10.3389/fcvm.2024.1504298

**Published:** 2024-10-15

**Authors:** Frontiers Editorial Office

A Retraction of the Case Report article Coronavirus disease 2019 (COVID-19) modified RNA vaccination-induced adult-onset still’s disease with fulminant myocarditis as initial presentation By Zhou M, Wong CK, Yeung WYW, Siu CW and Yin L (2023). Front. Cardiovasc. Med. 10:1066699. doi: 10.3389/fcvm.2023.1066699

Following publication, concerns were raised regarding the duplication of patient data in the article cited above in Frontiers in Cardiovascular Medicine and Hong Kong Medical Journal Kan A, Yeung W, Lau CS, Li P (2023) Adult-onset Still's disease after mRNA COVID-19 vaccination presenting with severe myocarditis with acute heart failure and cardiogenic shock: a case report Hong Kong Med J. 2023 Apr;29(2):162–4 | Epub 20 Mar 2023 https://doi.org/10.12809/hkmj2210110.

An investigation was conducted in accordance with Frontiers' policies, whereby it was confirmed that duplicate publication had occurred and the article has been retracted.

This retraction was approved by the Specialty Chief Editor of Cardiovascular Medicine. The author(s) agree to this Retraction.

